# Medicinal chemistry advances in targeting class I histone deacetylases

**DOI:** 10.37349/etat.2023.00166

**Published:** 2023-08-31

**Authors:** Diaaeldin I. Abdallah, Elvin D. de Araujo, Naman H. Patel, Lina S. Hasan, Richard Moriggl, Oliver H. Krämer, Patrick T. Gunning

**Affiliations:** Sun Yat-sen University, China; ^1^Department of Chemical & Physical Sciences, University of Toronto Mississauga, Mississauga, Ontario L5L 1C6, Canada; ^2^Department of Chemistry, University of Toronto, Toronto, Ontario M5S 2E8, Canada; ^3^Institute of Animal Breeding and Genetics, University of Veterinary Medicine, 1210 Vienna, Austria; ^4^Department of Toxicology, University of Mainz Medical Center, 55131 Mainz, Germany

**Keywords:** Histone deacetylases, zinc-binding group, cap group, epigenetic regulation, small-molecule inhibitors, medicinal chemistry

## Abstract

Histone deacetylases (HDACs) are a class of zinc (Zn)-dependent metalloenzymes that are responsible for epigenetic modifications. HDACs are largely associated with histone proteins that regulate gene expression at the DNA level. This tight regulation is controlled by acetylation [via histone acetyl transferases (HATs)] and deacetylation (via HDACs) of histone and non-histone proteins that alter the coiling state of DNA, thus impacting gene expression as a downstream effect. For the last two decades, HDACs have been studied extensively and indicated in a range of diseases where HDAC dysregulation has been strongly correlated with disease emergence and progression—most prominently, cancer, neurodegenerative diseases, HIV, and inflammatory diseases. The involvement of HDACs as regulators in these biochemical pathways established them as an attractive therapeutic target. This review summarizes the drug development efforts exerted to create HDAC inhibitors (HDACis), specifically class I HDACs, with a focus on the medicinal chemistry, structural design, and pharmacology aspects of these inhibitors.

## Introduction

**Figure 1:**
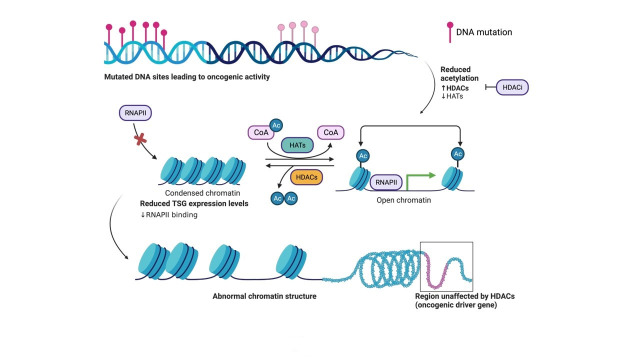
The basic principle of acetylation status for open (euchromatin) or closed chromatin (heterochromatin). Structural organization of DNA wrapped around histone proteins, and the effects on expression of proteins such as TSG transcription due to the downregulation of HATs and upregulation of HDACs during oncogenic activity in the cell. Acetylation of histones on surface exposed Lys residues by HAT. Histone acetylation yields an amide that is neutral at physiological pH and thus weakly interacts with the negatively charged DNA, thus allowing the cellular transcription machinery to access TSGs for transcription. Deacetylation of histones by HDAC. Histone deacetylation yields a primary amine that is positively charged at physiological pH and thus strongly interacts with the negatively charged DNA, thus preventing the cellular transcription machinery (ie. RNAPII) from accessing TSGs for transcription. HDACi aid in the inhibition of HDAC activity. The figure was created with Biorender.com. RNAPII: RNA polymerase II; CoA: coenzyme A; Ac: acetyl; HDACi: HDAC inhibitor

Histone deacetylases (HDACs) are enzymes that belong to a class of hydrolases that catalyze the removal of acetyl groups from lysine (Lys) residues on histones and non-histone substrates (Figure 1). Lys acetylation is frequently dysregulated in several indications such as autoimmune diseases or cancers, due to post-translational modification of acetylation changing protein stability/activity status. Acetylation functions as a mode for epigenetic regulation, best known for histone acetylation as a paradigm for tailored gene transcription machinery outcomes. Collectively, both histone acetyl transferases (HATs) and HDACs, play an important role in precisely tuning the balance of transcriptional activation and repression of the human genome. Broadly, HATs are recruited by transcription factors and co-activators while HDACs are recruited by transcriptional repressors and co-repressors, highlighting the relevance of HDAC therapeutic targets in multiple disease indications, such as a wide spectrum of carcinomas or acute leukemia, lymphoma, or sarcomas where tumor suppressor gene (TSG) expression is often silenced, repressed or mutated to loss of function due to missense or stop codon mutations for example [1].

HDACs are stratified into 4 classes—I, IIA, IIB, and IV (Table 1). These HDACs are zinc (Zn)-dependent due to the presence of a Zn^2+^ ion at the base of their catalytic pocket. Class I HDACs are comprised of HDACs 1, 2, 3, and 8, with the remaining classes indicated in Table 1. There also exists an independent class of HDACs (class III) which is known as sirtuins, which are nicotinamide adenine dinucleotide (NAD^+^)-dependent. These enzymes are also present in the mitochondria due to their involvement in an array of metabolic pathways. The grouping of HDACs into their respective classes is based on their homology to yeast transcriptional regulators [2]. However, the individual HDAC numbering refers to their date of discovery.

**Table 1:** Zn-dependent HDAC protein classification data [1, 3]

**Class**	**HDAC**	**Localization**	**Representative PDB code**	**Numbers of amino acids**
I	1	Nuclear	4BKX	483
	2	Nuclear	3MAX	488
	3	Nuclear/cytoplasmic	4A69	428
	8	Nuclear	5VI6	377
IIA	4	Nuclear/cytoplasmic	5ZOO	1,084
	5	Nuclear/cytoplasmic	-	1,122
	7	Nuclear/cytoplasmic	3C0Y	912
	9	Nuclear/cytoplasmic	1TQE	1,069
IIB	6	Cytoplasmic	3PHD	1,215
	10	Cytoplasmic	6WDY	669
IV	11	Nuclear	-	347

-: not applicable; PDB: Protein Data Bank

Class I HDACs are attractive therapeutic targets due to their involvement in multiple pathways that promote oncogenesis, diabetes, cardiac disorders, neurodegenerative diseases, etc. [4]. This chapter highlights recent advances in the HDAC-targeting drug candidates, with an emphasis on class I HDACs from a medicinal chemistry lens. Historically, selective HDAC inhibition has been challenging, and optimization through medicinal chemistry approaches has shown an improved outlook on the future of HDACi design.

## HDACi engage the catalytic pocket to sterically block the catalytic activity

### Architecture of HDAC catalytic tunnel

**Figure 2:**
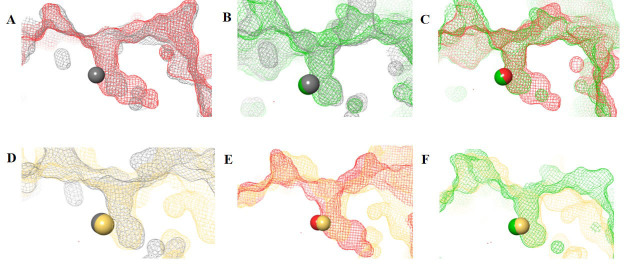
HDAC catalytic tunnel architecture. (A) Catalytic tunnels of HDAC1 (grey) and HDAC2 (red) superimposed; (B) catalytic tunnels of HDAC1 (grey) and HDAC3 (green) superimposed; (C) catalytic tunnels of HDAC2 (red) and HDAC3 (green) superimposed; (D) catalytic tunnels of HDAC1 (grey) and HDAC8 (gold) superimposed; (E) catalytic tunnels of HDAC2 (red) and HDAC8 (gold) superimposed; (F) catalytic tunnels of HDAC3 (red) and HDAC8 (gold) superimposed. Zn atoms are represented as spheres with the corresponding protein mesh surface colors. All images were generated via Maestro according to the PDB codes as detailed in Table 1

The HDAC catalytic tunnel represents a slim groove that can accommodate acetyl-Lys to undergo deacetylation [5]. The residues around the catalytic tunnel tend to be highly conserved among the class I HDACs, particularly HDACs 1–3 (Figure 2A–E). However, HDAC2 has a deeper catalytic tunnel that extends further into the protein when compared to HDACs 1 and 3. On the other hand, HDAC3 and HDAC8 have smaller catalytic tunnels without additional room in the lower periphery (Figure 2F). One of the major challenges, however, in targeting HDACs 1–3 is due to their existence in multimeric states and they are often complex with other co-factors that perform different functions [6]. HDAC8 is unique within the HDAC family, and while it is structurally the most similar to other class I HDAC catalytic domains it operates in a monomeric fashion [7]. Hence, HDAC3 shares the most similarity between HDAC1 and HDAC8, while HDAC1 shares the most similarity between HDAC2 and HDAC3, and HDAC2 and HDAC8 are the most distant structurally (Figure 2D and 2E).

### HDACi pharmacophore

**Figure 3:**
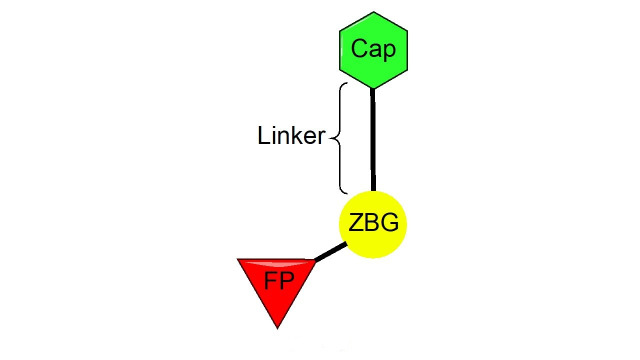
General class I HDACi pharmacophore

The vast majority of HDACi developed to date have exploited the Zn^2+^ center in the catalytic tunnel of HDACs by employing an electron-rich moiety commonly known as a ZN-binding group (ZBG) [8]. Other differences such as the catalytic tunnel residues and protein surface residues have led to diversity in inhibitor design to leverage selectivity among different HDAC isozymes. Consequently, HDACi has distinct regions, each interacting with a specific part of the HDAC catalytic tunnel. As such, the HDACi pharmacophore consists of 4 main portions (Figure 3)—(A) the ZBG, which coordinates the Zn^2+^ at the base of the catalytic tunnel; (B) a linker that interacts with the residues on the sides of the catalytic tunnel; (C) a cap group, which interacts with the surface residues around the rim of the catalytic tunnel, and in the case of class I HDACi; and (D) foot-pocket (FP) group, which interacts with the lower periphery of the catalytic tunnel, is seen in many examples of HDAC1 and HDAC2 inhibitors since their tunnels extend deeper into the protein core [8].

HDACi selectivity is traditionally gained from optimizing the cap group due to the conservation of residues around the interior of the catalytic tunnel with increased variation around the surface residues of each HDAC [7].

### Structural differences among HDAC family members allow for different ZBG chelation patterns and selectivity

**Figure 4:**
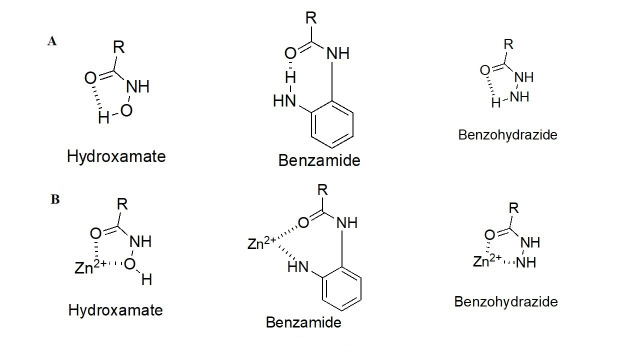
HDACi pharmacophore and chemotypes with juxtaposition to the critical Zn^2+^ ion. (A) Different chemotypes of ZBGs used to target class I HDACs with intramolecular hydrogen bonding patterns; (B) ZBGs coordinating to Zn^2+^

As detailed above, the ZBG enthalpically drives the HDAC target engagement. While most HDACi tend to employ a hydroxamic acid as a ZBG, class I HDACs (particularly HDACs 1–3) tend to be the only class of HDACs inhibited by 2-amino anilides as a ZBG (Figure 4A) [9–13]. This may be attributed to a more spacious compartment at the base of their catalytic tunnels that can accommodate the larger 2-amino anilide group as compared to the smaller hydroxamic acid. In many cases, reports of benzohydrazides have also been employed as ZBGs for targeting class I HDACs [14–16]. Although certain molecules did exhibit substantial selectivity towards class I HDACs, there seem to be no clear patterns that definitively favor the use of benzohydrazides for HDACs 1–3 over other HDACs (e.g., HDAC6 and HDAC8).

While intramolecular hydrogen bonding is possible within a hydroxamic acid, a stronger, more geometrically favorable intramolecular hydrogen bond can occur within the 2-amino anilide moiety (Figure 4B) [17]. However, because of the intra- to inter-molecular conversion, 2-amino anilides have been reported to exhibit slow-binding kinetics with HDACs 1–3. For example, a selective HDAC3 inhibitor, RGFP966, has been shown to exhibit a time-dependent reduction in half maximal inhibitory concentration (IC_50_) with longer pre-incubation times [18]. This time-dependent inhibition could be attributed to the two-step mechanism of the 2-aminoanilide binding to HDACs 1–3. Initially, the inhibitor orients in a fashion that is structurally complementary to the catalytic tunnel. Subsequently, the intramolecular hydrogen bond within the 2-amino anilide breaks to coordinate with the Zn (II) [17]. While slow, the interaction between the 2-amino anilide forms a much tighter interaction with the HDAC proteins in comparison to the fast binding of hydroxamates which is often coupled to a faster elimination and poorer drug pharmacokinetic (PK) profile. Hence, 2-amino anilides tend to have significantly longer residence times in the protein than hydroxamic acids and this unlocks unique binding profiles of the 2-amino anilide inhibitors [17, 19, 20].

## Current class I HDACis in the literature

Here, drugs target class I HDACs, clinical candidates, as well as a selection of upcoming inhibitors with potency and selectivity towards HDACs 1–3, or other HDACs.

### Food and Drug Administration-approved inhibitors

To date, there have been several HDACis approved for clinical use [21] which include hydroxamic acids, 2-amino anilides, and thiol-based inhibitors (a disulfide bridge that is reduced in the intracellular environment). Most of the approved inhibitors operate in a pan-inhibitory fashion, which engages all/most of the 11 HDACs with limited gene product selectivity (Tables 2 and 3). However, some of the Food and Drug Administration (FDA)-approved inhibitors exhibit class-selective target engagement. For example, romidepsin engages all classes of HDACs apart from class IIA. Contrastingly, tucidinostat, a 2-amino anilide, engages exclusively in class I HDACs with limited inhibition of HDAC10 and HDAC11.

**Table 2:** Structures of clinically approved HDAC inhibitors

**Name**	**Structure**
Vorinostat	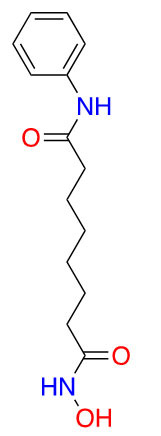
Tucidinostat	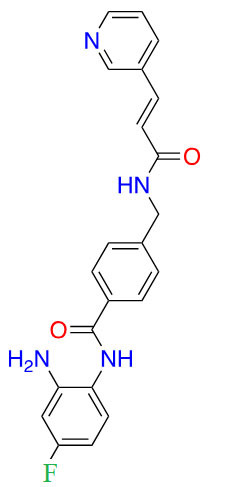
Romidepsin	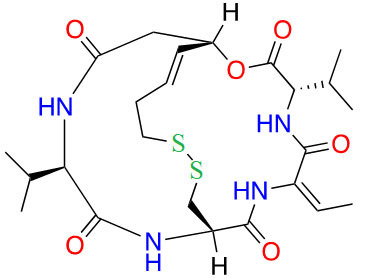
Panobinostat	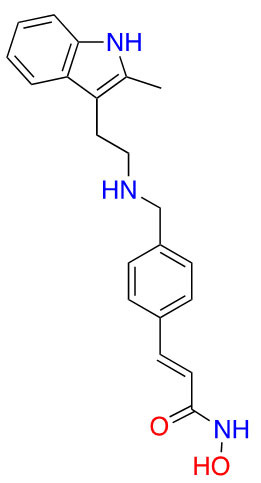
Belinostat	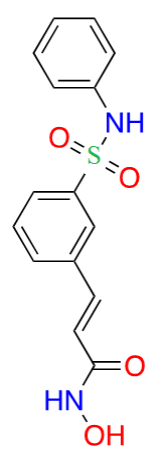

**Table 3:** Clinically approved HDAC IC_50_ values against all *HDAC* gene products [22]

**HDAC isozyme**	**Vorinostat, hydroxamate (nmol/L**)	**Tucidinostat, anilide (nmol/L**)	**Romidepsin, thiol (nmol/L**)	**Panobinostat, hydroxamate (nmol/L**)	**Belinostat, hydroxamate (nmol/L**)
1	60	100	1	3	26
2	42	200	1	2	22
3	36	100	1	2	19
4	20	> 10,000	647	1	15
5	36	> 10,000	> 1,000	1	25
6	29	> 10,000	226	1	10
7	129	> 10,000	> 1,000	2	51
8	173	700	> 1,000	22	22
9	49	> 10,000	> 1,000	1	24
10	60	100	1	31	59
11	31	400	0.3	4	27

### Inhibitors in clinical trials

With regards to class I HDACi, there are eight inhibitors currently undergoing clinical trials which are class I selective.

Out of the eight class I selective HDACi in clinical trials—three are 2-amino anilides; four are hydroxamic acids; and one is a thiol (Table 4).

**Table 4:** Class I HDACis undergoing clinical trials [23]

ZBG class	Name	Structure	Clinical trial phase	Indications
Benzamide	Entinostat	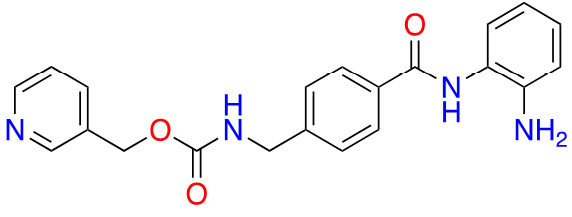	2	Breast cancer [24] and elanoma [25]
	RDN-929	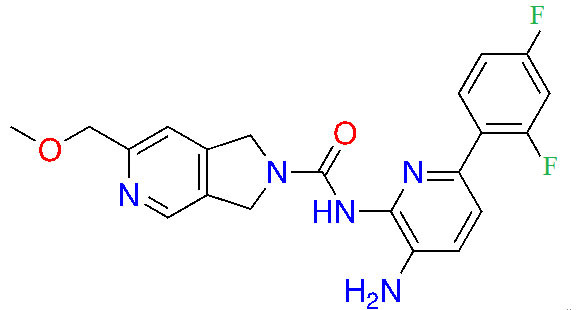	1	Healthy patients [26]
	Mocetinostat	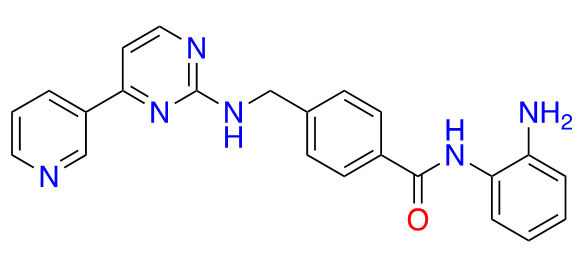	1	Lymphoma [27] and lung cancer [28]
Hydroxamic acid	Pracinostat	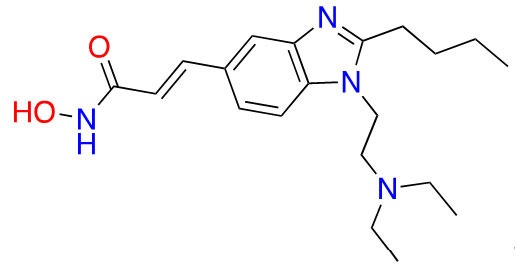	2	AML [29]
	Resminostat	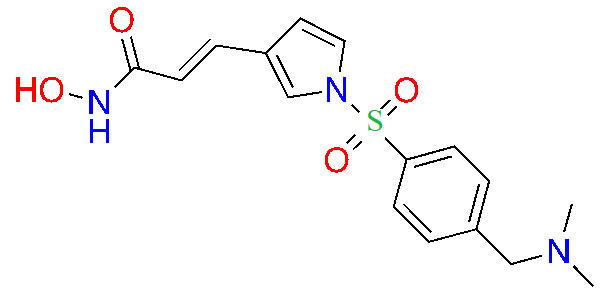	1/2	Colorectal CTCL [30]
	Givinostat	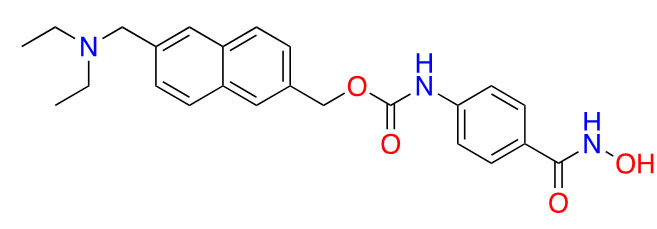	2	Polycythemia vera [31], DMD [32], and arthritis [33, 34]
	Remetinostat	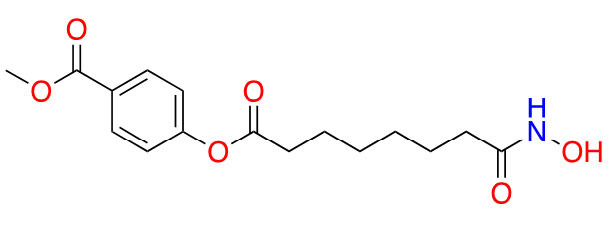	2	CTCL [35]
Thiol	OKI-179	Structure undisclosed	1	Solid tumors [36]

AML: acute myeloid leukemia; CTCL: cutaneous T-cell lymphoma; DMD: duchenne muscular dystrophy

### Advanced preclinical HDACis

Highlighted below is a selection of inhibitors that are in the advanced preclinical stages of assessments, based on ZBGs. An analysis of their chemical and structural features and their contributions to both potency and selectivity against HDAC protein family members is provided.

#### Hydroxamic acids

**Figure 5:**
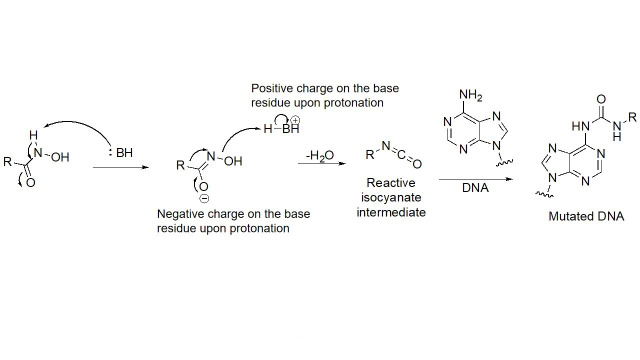
Lossen’s rearrangement. The mechanism through a chemical reaction called Lossen’s rearrangement is depicted, exemplified by a general hydroxamate through which the isocyanate intermediate forms, causing DNA mutagenesis and eventual toxicity [41]

Due to its superior Zn^2+^ binding affinity, hydroxamic acids are the most common ZBG in HDACi, as demonstrated by their abundance among clinical and approved inhibitors. As a polar moiety with increased capacity for both inter- and intra- molecular hydrogen bonds, hydroxamates exhibit excellent solubility and *in vitro* stability [37]. Despite their exquisite potency against HDACs, installing a hydroxamate on any molecule with limited optimization is strongly biased for generating pan-HDACi, among many other possible interactions with metalloproteins (aminopeptidases, carbonic anhydrase, etc.) that can be chelated in a similar manner [38]. Hence, hydroxamates are often faced with selectivity barriers, which are mitigated via optimization of the linker and cap groups [39]. Another major drawback to hydroxamates is their poor PKs due to rapid elimination and metabolite cytotoxicity, thus decreasing their potential for clinical use [40]. Cytotoxicity mainly occurs through a Lossen’s rearrangement (Figure 5) where a reactive isocyanate intermediate forms and can interact with DNA, inducing mutagenesis [41].

**Figure 6:**
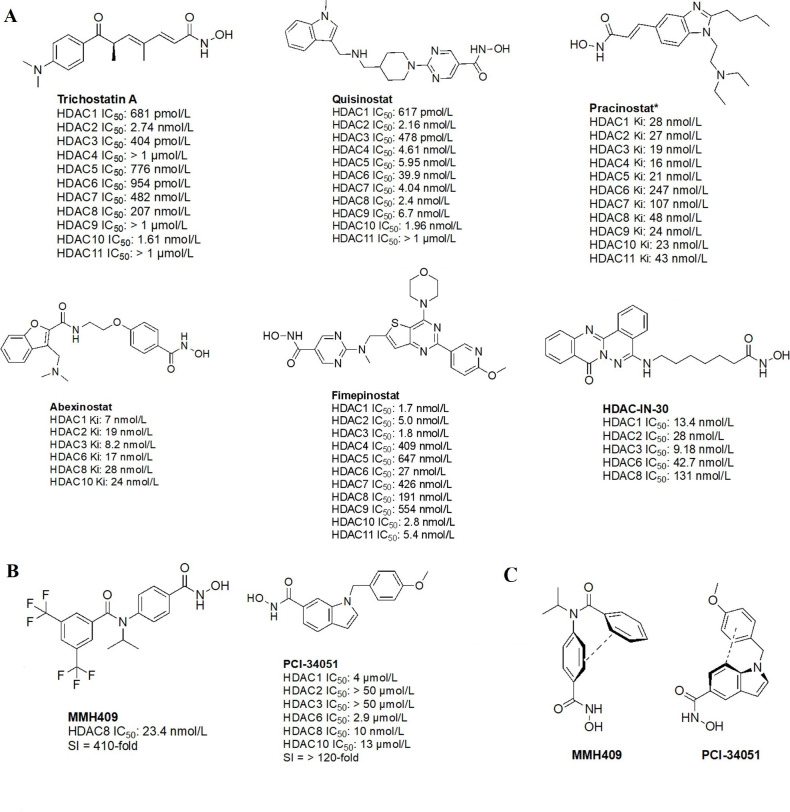
Pan-HDACi overview. (A) Broadly acting pan-HDACs bearing a hydroxamate ZBG that exhibit target engagement towards class I HDACs; (B) inhibitors bearing a hydroxamate ZBG that are HDAC8-selective [42–49]; (C) potential “*cis*” conformations adopted by HDAC8-selective inhibitors [43]. ^*^ Asterisks refer to inhibitors that are either clinical candidates or FDA-approved drugs. SI: selectivity index; Ki: inhibition constant

The majority of hydroxamate HDACi have a propensity to act in a pan-inhibitory fashion (Figure 6A) [42–49]. For example, trichostatin A displays picomolar potency towards class I HDACs but also inhibits class IIB with nanomolar potency [43]. This phenomenon is also observed with abexinostat (PCI-24781), a preclinical HDACi developed for the treatment of B-cell lymphoma, and the more recently developed HDAC-IN-30 [47, 49, 50]. Notably, hydroxamates inhibit HDAC6 preferentially over other HDACs, whereas class I inhibition is a result of extensive optimization of the inhibitor cap region to achieve higher affinity for HDACs 1–3 and HDAC8 [8, 51–54].

Notably, linker length and composition are not directly correlated with regard to class or isozyme selectivity. For example, trichostatin A contains a chain of sp^2^-hybridized carbons for a linker while fimepinostat and quisinostat contain pyrimidines as linkers, yet all 3 molecules inhibit HDACs 1–3 in the picomolar range (Figure 6A) [43, 44, 48]. Similarly, pracinostat and abexinostat display nanomolar potencies towards class I and class IIB HDACs in spite of bearing different linkers—cinnamyl and phenyl respectively [42, 43].

To further confirm the lack of linker significance in HDAC isozyme selectivity, two HDAC8-selective inhibitors, MMH409 and PCI-34051 (Figure 6B) show comparable IC_50_ values (23.4 nmol/L and 10 nmol/L respectively) while containing an anilide and an indole as linkers. A unique feature of these HDAC8 selective inhibitors is their ability to go through an intramolecular π–π stack (Figure 6C). In the case of MMH409, density functional theory (DFT) calculations have shown that the molecule adopts a *cis*:*trans* ratio of 93%:7% in solution, which when docked computationally, was shown to be the most stable binding orientation to HDAC8 [43]. While not confirmed for PCI-34051, the molecule can adopt a similar conformation which could explain its selectivity towards HDAC8.

Interestingly, the largest diversities between HDACi are depicted within the cap group where differences in the surface residues of HDAC isozymes are optimized for substrate recognition, unlike the conserved residues around the catalytic tunnel. An interesting observation is the presence of free-amine and *N*-heterocycles in the cap region across multiple inhibitors (e.g., fimepinostat, quisinostat, HDAC-IN-30, and pracinostat), which may be contributing to increased affinity towards HDAC. This is because the presence of several aspartate residues around the rim of the catalytic tunnels (such as adjacent Asp92 and Asp93 in HDAC3) allows for hydrogen bonding opportunities and potential salt bridges formation depending on the ionization state of heterocycles and amines in the local pH environment.

#### Ortho-amino-anilides

**Figure 7:**
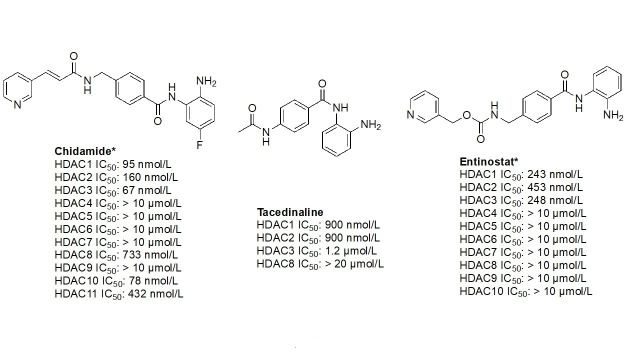
Examples of class I HDACi [55–57]. ^*^ Asterisks refer to inhibitors that are either clinical candidates or FDA-approved drugs

**Figure 8:**
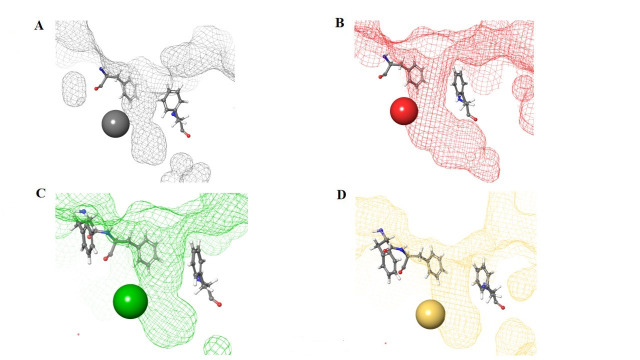
Structural presentation of class I HDAC catalytic centers. Class I HDAC tunnels surrounded by hydrophobic Phe residues. (A) HDAC1 (grey mesh) surrounded by Phe150 (in front of the tunnel in the image) and Phe205 (behind the tunnel in the image); (B) HDAC2 (red mesh) surrounded by Phe155 (in front of the tunnel in the image) and Phe210 (behind the tunnel in the image); (C) HDAC3 (green mesh) surrounded by Phe144 (in front of the tunnel in the image) and Phe199 and Phe200 (behind and to the side of the tunnel in the image); (D) HDAC8 (gold mesh) surrounded by Phe152 (in front of the tunnel in the image), Phe207, and Phe208 (behind and to the side of the tunnel in the image). All images in this figure were generated by Maestro

**Figure 9:**
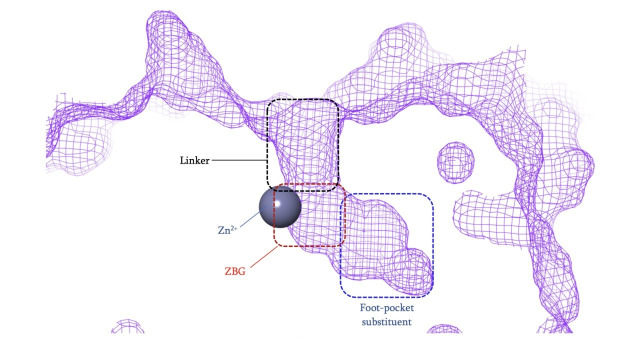
Structural presentation of the HDAC2 catalytic center. HDAC2 catalytic tunnel (PDB: 3MAX) labeled with HDACi binding moieties [58, 59]

**Figure 10:**
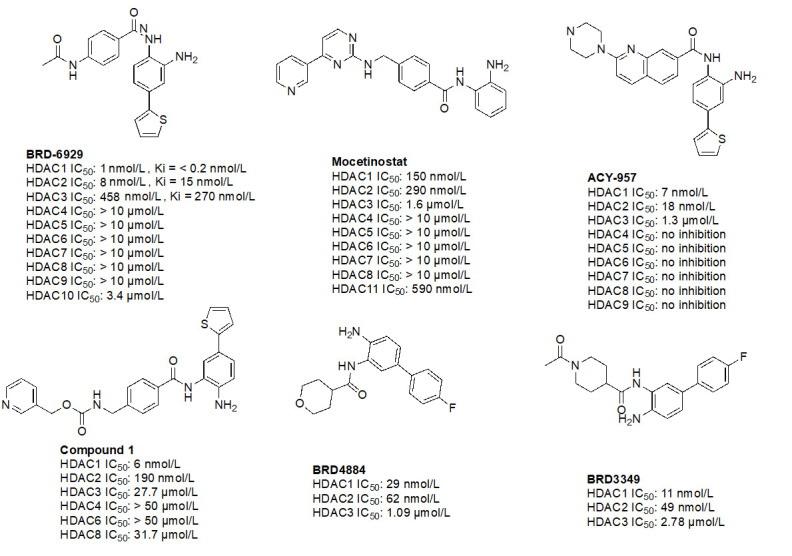
Examples of selective HDAC1/2 inhibitors [55, 60–64]

**Figure 11:**
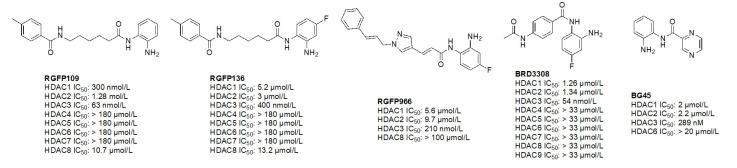
Examples of selective HDAC3 inhibitors [65–69]

Due to the advantage of a bigger catalytic tunnel of class I HDACs, 2-amino anilides are often exploited for class I HDAC selectivity as they are larger in comparison to hydroxamates (Figure 4). Therefore, hydroxamate toxicity [via isocyanate formation and eventual mutagenicity (Figure 5)] and persistent off-target effects are eliminated. Surprisingly, despite being selective for class I, 2-amino anilides have a very limited affinity towards HDAC8 (Figure 7). An additional benefit of employing 2-amino anilides is the presence of a phenyl ring which can potentially engage in π–π interactions within the tunnel residues [e.g., phenylalanine (Phe) as demonstrated in Figure 8] further engaging the HDAC protein. Additionally, the ring can be both sterically and electronically tuned for a stronger interaction with individual HDAC family members. For example, the addition of an FT substituent (usually a heterocycle at the 5-position of the anilide), improves the selectivity profile for HDAC2 due to occupation of the lower periphery of the HDAC2 pocket (Figure 9). Thus, inhibitors bearing a FP substituent will not inhibit HDAC3 but will selectively inhibit HDAC1 and HDAC2 (Figure 10). Another notable feature of HDAC3-selective *O*-amino anilides is the presence of fluorine at the 4- or 5- position of the phenyl ring [e.g., tucidinostat (Table 2), RGFP966, and RGFP109 (Figure 11)]. While the size of the HDAC3 catalytic tunnel is not amenable to anilide substituents larger than fluorine atoms, this trend has been replicated through multiple structure-activity relationship (SAR) studies.

##### Class I selective *O*-amino anilides

The functional groups, 2-amino anilides, are often used as the golden standard for targeting HDACs 1–3 due to their exclusivity of binding that is not observed with hydroxamates, or any other ZBG. Most linkers contain a benzamide unit [e.g., chidamide, entinostat, and tacedinaline/CI994 (Figure 7)] linked at the carbonyl end of the anilide [55–57]. Given that the natural substrate for HDACs includes an acetyl-Lys tethered to a hydrophobic chain, it is reasonable to design drugs bearing a hydrophobic linker. While some class I HDACis bear alkyl linkers [e.g., RGFP109 and RGFP106 (Figure 11)], most class I HDACi contain benzamide due to favorable interactions within the Phe residues in the hydrophobic tunnel [58].

For example, the tunnel of HDAC1 is sandwiched between two Phe residues [Phe150 and Phe205 (Figure 8A)], which creates favorable π–π stacks with benzamides in inhibitors [58]. Similarly, HDAC2, due to structural similarities with HDAC1, shares the same Phe residues [Phe155 and Phe210 (Figure 8B)] in identical locations around the tunnel [58]. In the same vain, HDAC3 and HDAC8 contain hydrophobic Phe residues that surround the tunnel. However, due to the closer similarity between HDAC3 and HDAC8, the tunnel is surrounded by 3 Phe residues [Phe144, Phe199, and Phe200 for HDAC3 (Figure 8C) and Phe152, Phe207, and Phe208 for HDAC8 (Figure 8D)] [58].

##### HDAC1/2 selective *O*-amino anilides

Given the presence of a lower cavity in the tunnel of HDAC1 and HDAC2, this is often exploited in drug design to delineate HDAC1 and HDAC2 from HDAC3. Substituting a heterocycle at the 5-position (meta) to the 2-amino anilide has been shown to significantly increase the potency of inhibitors for HDAC1 and HDAC2 while remarkably disengaging HDAC3, which is due to the occupation of the newly introduced heterocycle at the “FT” in the lower periphery of the HDAC tunnel [e.g. HDAC2 (Figure 9)] [8]. A further example is an entinostat (Figure 7) as a pan-class I HDACi [19]. However, when substituted with a 2-thiophene group at the 5-position of the anilide ring as seen in compound 1 (Figure 10), it becomes > 4,600-fold selective for HDAC1 over HDAC3 and > 145-fold selective for HDAC2 over HDAC3 [60]. This example has also been reproduced with multiple other inhibitors such as BRD-6929, BRD-4884, BRD3349, and ACY-957 [61–63]. In the case of BRD-6929 and ACY-957, the substitution involved a thiophene group, which has emerged as the most commonly employed method to induce HDAC1/2 selectivity [61, 63]. Note that BRD-6929 is a derivative of tacedinaline, which, without the thiophene substitution, is a pan-class I inhibitor in the micromolar range [61]. Admittedly, the substitution of the thiophene group on tacedinaline to yield BRD-6929 improves the potency against all 3 HDACs. However, BRD-6929 becomes HDAC1/2 selective with single-digit nanomolar potency [57, 61]. In other cases, such as BRD4884 and BRD3349, HDAC1/2 selectivity was still achieved over HDAC3 despite the substitution being of a 4-fluorophenyl group on the anilide, not thiophene [62]. While BRD4884 and BRD3349 (Figure 10) do not possess a cap group moiety, the differences in the linker (4-tetrahydrofuranyl *vs.* 4-(*N*-acetyl)-piperidinyl) do not change the *in vitro* activity of the molecules dramatically [62]. Finally, an example of the importance of the cap group is highlighted in mocetinostat (Figure 10), where selectivity is gained for HDAC1/2 against HDAC3 (5.5–10.7 fold selective) without resorting to the use of an FT substituent [64].

##### HDAC3 selective *O*-amino anilides

A common feature of HDAC3-selective molecules includes (A) a fluorine atom at the para-position of anilide, (B) a vinyl group in the linker, and (C) *N*-heterocycles in the linker towards the cap group. For this reason, RGFP966 (Figure 11) has emerged as the most frequently employed tool for HDAC3 pharmacologic inhibition in biological studies [9, 13, 67, 69–71]. A notable comparison between RGFP109 and RGFP136 (Figure 11) shows that despite the potency cost incurred (63 nmol/L to 400 nmol/L for HDAC3), when a class I HDACi (RGFP109) is substituted with fluorine at the para position of the ZBG, it becomes an HDAC3 selective molecule [RGFP109 IC_50 (HDAC3)_ = 63 nmol/L; RGFP136 IC_50 (HDAC3)_ = 400 nmol/L]. Similarly, comparing tacedinaline (Figure 7) and BRD3308 (Figure 11) reveals that installing fluorine at the para position of the ZBG significantly improves the selectivity for HDAC3 (23–25-fold improvement) despite acting in a pan-class I inhibitory manner prior to the fluorine substitution. Finally, a cap-less inhibitor, BG45, bears a pyrazine linker, which allows for faster kinetics of binding to the target protein due to the rigidity of the molecule and lack of degrees of freedom that comes with a cap group [65].

**Figure 12:**
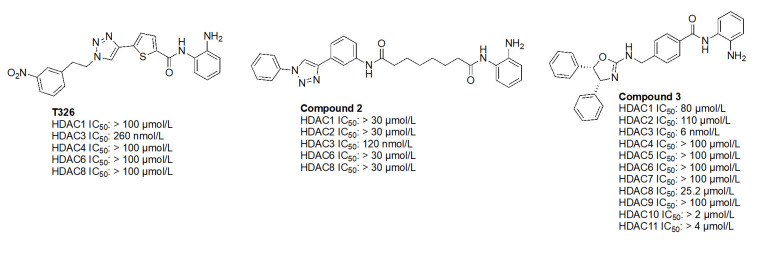
Examples of HDAC3 selective inhibitors [72–74]

**Figure 13:**
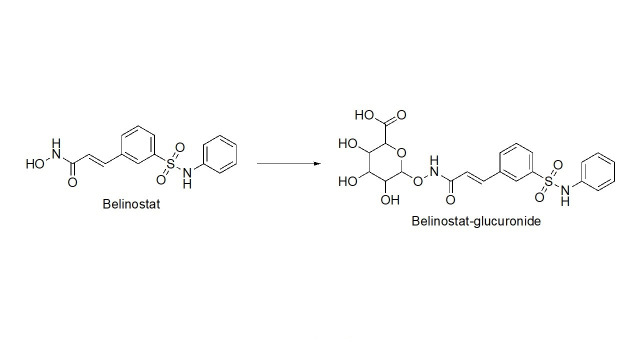
Overview of the chemical reaction of belinostat transformation. Transformation of belinostat to belinostat glucuronide in phase II metabolism [75–77]

While the molecules mentioned in Figure 11 are the most studied in the literature for HDAC3 selectivity, some molecules have been published with exquisite (250–385-fold) HDAC3 selectivity and have yet to be pursued in further biological studies (Figure 12) [72, 73]. For example, T326 and compound 2 (Figure 12) take advantage of installing conjugated tricyclic systems in the same molecule and utilize π–π stacking and hydrophobicity interactions (Figure 8) [72, 73]. T326 is comprised of anilide as the ZBG, followed by a thiophene, and a triazole [73] while compound 2 involves a conjugated phenyl-triazole-phenyl as the cap group [72]. Further reaffirming the *N*-heterocycle trend observed with HDAC3 selective inhibitors, both T326 and compound 2 bear triazole functionalities [72, 73]. Finally, the importance of the cap group was further highlighted in the discovery of compound 3 [fold selective for HDAC3 (Figure 12 and Figure 13)] where substitution patterns and stereochemistry of the cap group dramatically influenced HDAC protein class member selectivity [74].

As discussed earlier (Structural differences among HDAC family members allow for different ZBG chelation patterns and selectivity), an interesting feature observed with class I HDACis, particularly 2-amino anilides, is slow on/off binding kinetics with the target protein [18]. Given the 2-amino anilide’s steric bulk, binding to the ZBG requires a two-step mechanism—(A) orientation of the drug and protein in a complimentary fashion and (B) breakage of the intramolecular hydrogen bond of the amino (NH_2_) and carbonyl (C=O) oxygen to chelate the Zn^2+^ [55]. The requirement for a multi-step process slows down the kinetics of binding since the protein must move dynamically to accommodate the larger ZBG (in comparison to a “thinner” hydroxamate or hydrazide) [55]. While the “on” rate of binding is slow, so is the “off” or dissociation rate. Through this study, it was found that for RGFP966, a selective HDAC3 inhibitor, the selectivity diminishes to the apparent 2-fold after 2 h of pre-incubation of the drug with the protein, allowing the binding to occur, and for an equilibrium to be achieved [18]. Hence, the kinetics of binding is important in HDAC family member inhibition, target engagement, and further downstream biological outcomes.

#### Benzohydrazides

Benzohydrazides have shown immense potential in targeting class I HDACs. Hydrazides hold a remarkable advantage over hydroxamates—avoidance of glucuronidation in phase II metabolism [75, 76]. Typically, UDP-glucuronosyl transferases (UGTs) transfer a glucuronide unit onto a hydroxyl unit to tag it for excretion in phase II metabolism (Figure 13), which was observed with the FDA-approved drug belinostat in clinical trials [75, 77]. This limits the utility of the drug due to faster elimination and deteriorates its PK profile.

**Figure 14:**
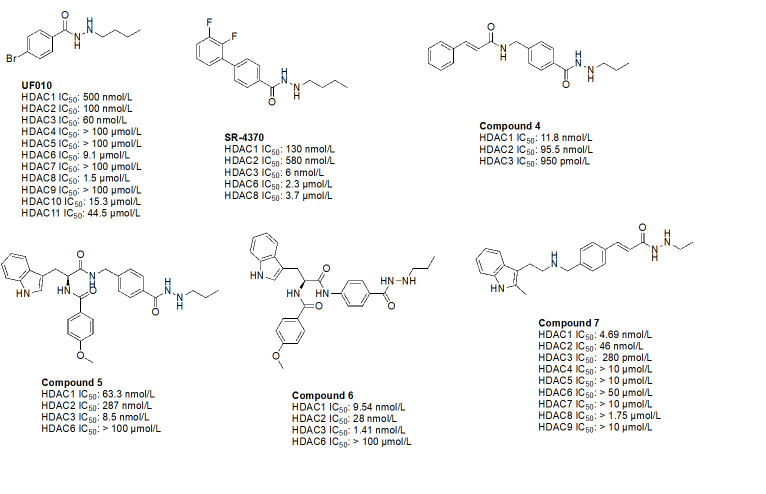
Examples of hydrazide HDACis [78–83]

The use of benzohydrazides has often been linked with moderate HDAC3 selectivity. Most hydrazides shown in Figure 14 bear an alkyl chain “tail” extending from the *β*-nitrogen of the hydrazide [78–82]. A propyl chain captures the ideal size of the alkyl chain to be installed as the linker. A butyl chain is also tolerated, although potency loss is observed across HDACs 1–3 with larger chains such as pentyl and larger groups [78–82].

UF010 (Figure 14) is a novel hydrazide inhibitor with a 4-bromophenyl as a linker with exquisite potency for class I HDACs, specifically HDAC2 and HDAC3 [81]. While UF010 has no cap group, the presence of the bromine atom, which is relatively larger in size, can act as a “plug” to the tunnel. The hydrazide moiety of UF010 bears a butyl chain (C4) tail. In a similar manner, SR-4370, a derivative of UF010 with a 2,3-difluorobenzene substituent in place of the bromine atom, shows a similar trend in potency, with improved selectivity towards HDAC3 (21.6–96.6-fold selectivity over HDAC2 and HDAC3) [82]. Compound 4, shares a similar structure to chidamide with the exception of the ZBG being a hydrazide instead of a fluoro-anilide, and the cap group containing a phenyl instead of a picolyl unit [56, 80]. This compound also bears a propyl chain extending from the hydrazides and offers higher potency and selectivity (12–100-fold) towards HDAC3 over HDAC2 and HDAC3 compared to chidamide (pan-class I HDACi) [56, 80]. Compound 5 and compound 6 display the importance of the cap group and its influence on potency [78]. For example, compound 6 shows an increase in inhibition of ~10-fold towards HDAC2, ~8-fold towards HDAC3, and ~7-fold increase towards HDAC1 when compared to compound 5, where the only difference between the two compounds is a methylene unit that makes the cap group of compound 5 an amine while compound 6’s cap group is a benzamide [78]. Finally, compound 7 is an example of a molecule that combines multiple moieties that yield selectivity for HDAC3—the presence of a hydrazide, cinnamyl linker, secondary amine at the end of the linker, an *N*-heterocycle (indole) in the cap group [79]. Compound 7 exhibits a 280 pmol/L potency towards HDAC3 with 16-fold selectivity over HDAC1 and > 160-fold selectivity over HDAC2 [79].

#### Thiols

**Figure 15:**
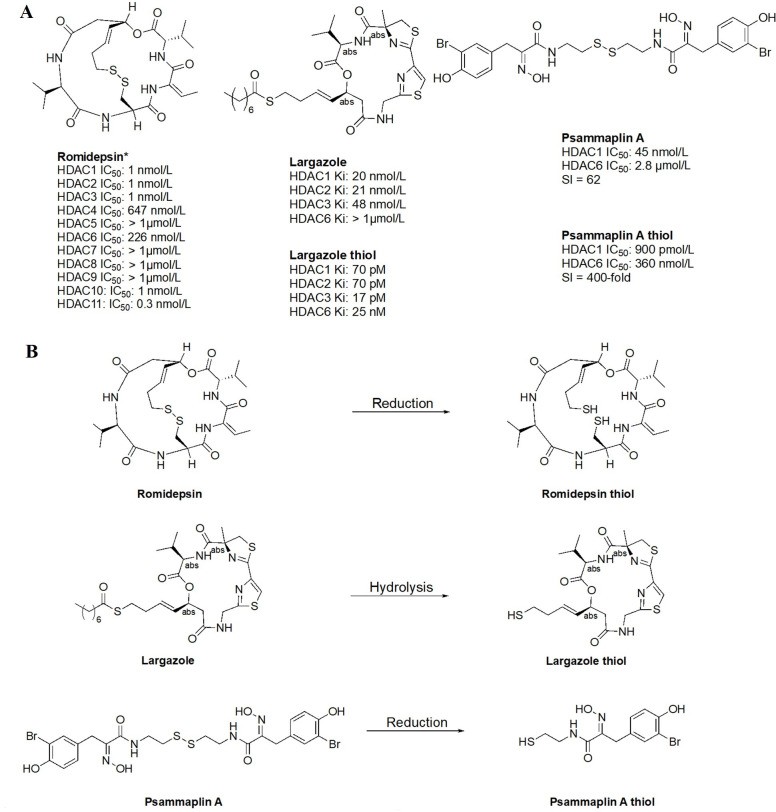
Examples of HDACi with thiols as ZBGs [84–87]. (A) IC_50_ values for enzymatic inhibition of different HDAC isozymes and (B) examples of processes that generate thiols out of their respective prodrugs (disulfide bridges/thioesters) [85]. ^*^ Asterisks refer to inhibitors that are either clinical candidates or FDA-approved drugs

Thiol inhibitors are often generated from disulfide bridges or thioesters that act as prodrugs, releasing the thiols as ZBGs upon metabolism. For example, romidepsin and psammaplin A (Figure 15A) contain disulfide bridges that are reduced in the intracellular environment to generate free thiols that are capable of Zn^2+^-chelation (Figure 15B) [84, 85]. Similarly, largazole (Figure 15A), a thioester, is recognized as a thioesterase substrate in cells and is hydrolyzed to release a free thiol as a ZBG (Figure 15B) [86, 87]. While offering superior potency towards HDACs, thiols tend to lack selectivity as seen in the example of romidepsin. However, in cellular contexts, the IC_50_ values of thiol HDACi decrease significantly, due to reduction/hydrolysis of prodrugs. Whereas *in vitro* experiments that lack reductants or hydrolases show increased IC_50_ values of such inhibitors [85, 86]. For example, largazole becomes a picomolar inhibitor upon hydrolysis, while psammaplin A also converts to a picomolar inhibitor upon reduction of the disulfide and allows for increased selectivity for HDAC1 (Figure 15A) [58–87].

### Novel ZBGs

In many cases, non-classical ZBGs (thiols, ketones, oxadiazoles, etc.) have been reported as inhibitors of HDACs [88, 89]. For example, trifluoromethyl oxadiazoles have been used to target other *HDAC* gene family products (class IIA HDACs).

**Figure 16:**
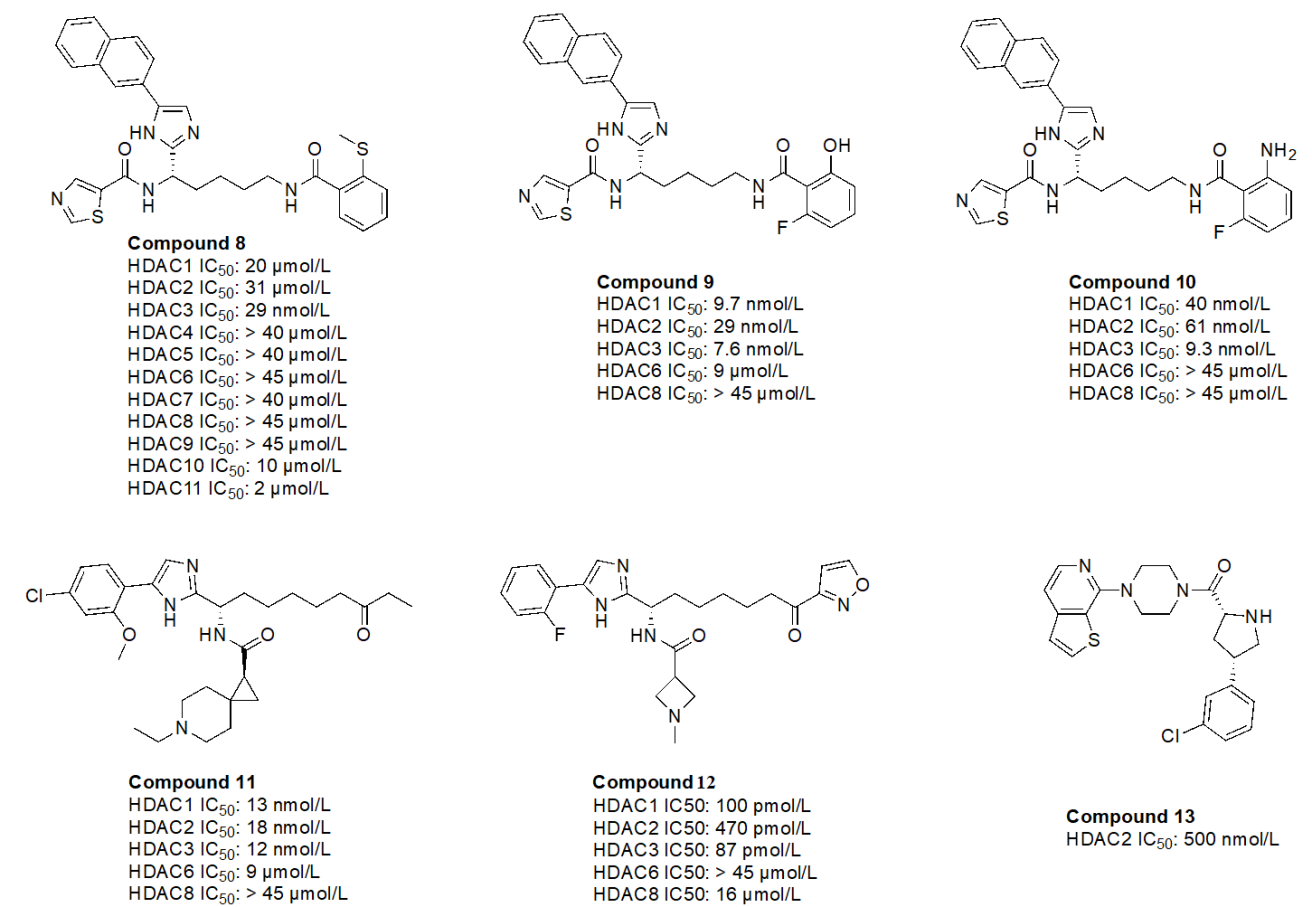
Examples of recently discovered novel ZBGs [88–91]

While *O*-amino anilides are treated as the golden standard for class I HDAC selectivity, Liu et al. [89] showed that Zn^2+^-binding can also occur by using novel ZBGs such as *O*-substituted benzamides (amide bond is transposed as opposed to anilides). As seen in the examples of compounds 8–10 (Figure 16), benzamides with ortho substituents selectively targeted class I HDACs [88, 89]. In particular, compound 8 was HDAC3 selective (> 300-fold) over any other class I HDAC [89]. This may be attributed to the strong chelation offered by the 2-methylthiobenzamide. Sulfur’s weak electronegativity, in addition to the inductive donation from the methyl group allows the thiol to operate as a stronger Lewis base that can chelate the Zn^2+^ in a more efficient manner [89]. With regards to compound 9 and compound 10, pan-class I inhibition was observed, likely due to the presence of 2-amino or 2-hydroxy substituents in the ZBG, giving a chelation pattern similar to classic 2-amino anilide ZBG (Figure 4B). Interestingly, compound 9 and compound 10 had a fluorine present at the second ortho position of the benzamide, suggesting the possibility of chemical tuneability of ZBGs as alluded to in Ortho-amino-anilides [89].

Compound 11, a ketone, has also been shown to exhibit class I HDAC selectivity with nanomolar potency [90]. While the linker and ZBG scaffolds of compound 11 could chelate any HDAC due to their similarity with acetyl-Lys, class I HDAC selectivity is likely observed due to optimization of the cap group, which further highlights the importance of cap group modifications that facilitate interactions with the surface residues of HDACs. On the other hand, compound 12, a derivative of compound 11 utilizes an acyl isoxazole as a ZBG [90]. Compound 12 has picomolar potency towards class I HDACs which is likely due to the improved optimization of the cap group, and the visibly close resemblance of the ZBG to hydroxamates, which are intrinsically quite potent against all HDACs.

Finally, compound 13, an orally active, brain-penetrant HDAC2 inhibitor, has recently been discovered through fragment-based drug discovery [91]. CNS penetrance is important for targeting neurological diseases such as Alzheimer’s disease where HDAC2 and HDAC3 activities are elevated [91]. Compound 13 has an FT group (chlorobenzene) that allows for HDAC2 selectivity. It also has an amino amide as a ZBG, which would likely have a similar chelation pattern to the Zn^2+^ as a 2-amino anilide (Figure 4B) where the carbonyl lone pair and amine lone pair coordinate to the Zn.

## Conclusions

HDACi has been studied extensively over the last 30 years and has come a long way since its inception. Despite the difficulty in attaining *HDAC* gene family member selectivity, recent efforts in the last ~15 years have been promising in achieving class selectivity among HDACs. Molecules discussed in this chapter are summarized below in Table 5 in terms of IC_50_ values against all 11 HDAC proteins. With regards to class I HDACi, the challenge remains to delineate HDAC1 and HDAC2 selectivity, whereas HDAC3 and HDAC8 seem to be somewhat distinguishable from the rest, albeit to a limited extent in the case of HDAC3. The general direction of class I HDACi seems to be accelerating in pursuit of optimizing 2-amino anilides since they offer the most class selectivity despite the reduced potencies. On the other hand, while hydroxamates offer exquisite potencies, they often lack HDAC family member selectivity and exhibit poor PKs which limits the range of their utility. Much like hydroxamates, ZBGs like benzohydrazides show promising potencies but these combinations in small molecules likely suffer from poor PK. The chemical space for novel ZBGs is expanding and shows promising results in the direction of specific HDAC protein member selectivity, which can open new therapeutic avenues to limit negative side effects or unwanted toxicity and to target specific HDAC disease-driving family members in cancer, autoimmunity, or chronic inflammatory diseases.

**Table 5:** Summary of all HDAC IC_50_ values

**Molecule name**	**HDAC protein members IC_50_ (mol/L)**										
	**1**	**2**	**3**	**4**	**5**	**6**	**7**	**8**	**9**	**10**	**11**
Vorinostat	0.06	0.042	0.036	0.02	0.036	0.029	0.129	0.173	0.049	0.060	0.031
Tucidinostat	0.1	0.2	0.1	> 10	> 10	> 10	> 10	0.7	> 10	0.1	0.4
Romidepsin	0.001	0.001	0.001	647	> 1	226	> 1	> 1	> 1	0.001	0.0003
Panobinostat	0.003	0.002	0.002	0.001	0.001	0.001	0.002	0.022	0.001	0.031	0.004
Belinostat	0.026	0.022	0.019	0.015	0.025	0.010	0.051	0.022	0.024	0.059	0.027
Trichostatin A	0.00068	0.0027	0.0004	> 1	0.776	0.00095	0.482	0.207	> 1	0.0016	> 1
Quisinostat	0.00062	0.002	0.00048	0.0046	0.006	0.0399	0.004	0.0024	0.0067	0.002	> 1
Pracinostat*	0.028	0.027	0.019	0.016	0.021	0.247	0.107	0.048	0.024	0.023	0.043
Abexinostat*	0.007	0.019	0.0082	-	-	0.017	-	0.028	-	0.024	-
Fimepinostat	0.0017	0.005	0.0018	0.409	0.647	0.027	0.426	0.191	0.554	0.0028	0.0054
HDAC-IN-30	0.0134	0.028	0.0092	-	-	0.0427	-	0.131	-	-	-
MMH409	-	-	-	-	-	-	-	0.0234	-	-	-
PCI-34051	4	> 50	> 50	> 50	-	-	2.9	0.010	-	13	-
Chidamide	0.095	0.160	0.067	> 10	> 10	> 10	> 10	0.733	> 10	0.078	0.432
Tacdeinaline	0.900	0.900	1.2	-	-	-	-	> 20	-	-	-
Entinostat	0.243	0.453	0.248	> 10	> 10	> 10	> 10	> 10	> 10	> 10	-
BRD-6929	0.001	0.008	0.458	> 10	> 10	> 10	> 10	> 10	> 10	3.4	-
Mocetinostat	0.150	0.290	1.6	> 10	> 10	> 10	> 10	> 10	-	-	0.590
ACY-957	0.007	0.018	1.3	NI	NI	NI	NI	NI	NI	-	-
Compound 1	0.006	0.190	27.7	> 50	-	> 50	-	31.7	-	-	-
BRD-4884	0.029	0.062	1.09	-	-	-	-	-	-	-	-
BRD-3349	0.011	0.049	2.78	-	-	-	-	-	-	-	-
RGFP109	0.300	1.28	0.063	> 180	> 180	>180	> 180	10.7	-	-	-
RGFP136	5.2	3	0.400	> 180	> 180	> 180	> 180	13.2	-	-	-
RGFP966	5.6	9.7	0.210	-	-	-	-	>100	-	-	-
BRD-3308	1.26	1.34	0.054	> 33	> 33	> 33	> 33	> 33	> 33	-	-
BG45	2	2.2	0.289	-	-	> 20	-	-	-	-	-
T326	> 100	-	0.260	>100	-	> 100	-	>100	-	-	-
Compound 2	> 30	>30	0.120	-	-	> 30	-	> 30	-	-	-
Compound 3	0.080	0.110	0.006	> 100	> 100	> 100	> 100	0.0252	> 100	> 2	> 4
UF010	0.500	0.100	0.060	> 100	> 100	9.1	> 100	1.5	> 100	15.3	44.5
SR-4370	0.130	0.580	0.006	-	-	2.3	-	3.7	-	-	-
Compound 4	0.0118	0.0955	0.00095	-	-	-	-	-	-	-	-
Compound 5	0.0633	0.287	0.0085	-	-	> 100	-	-	-	-	-
Compound 6	0.00954	0.028	0.00141	-	-	> 100	-	-	-	-	-
Compound 7	0.00469	0.046	0.00028	> 10	> 10	> 50	> 10	> 1.75	> 10	-	-
Largazole*	0.020	0.021	0.048	-	-	> 1	-	-	-	-	-
Largazole thiol*	0.00007	0.00007	0.000017	-	-	0.025	-	-	-	-	-
Psammaplin A*	0.045	-	-	-	-	2.8	-	-	-	-	-
Psammaplin A thiol	0.0009	-	-	-	-	0.360	-	-	-	-	-
Compound 8	20	31	0.029	> 40	> 40	> 45	> 40	> 45	> 45	10	2
Compound 9	0.0097	0.029	0.0076	-	-	9	-	> 45	-	-	-
Compound 10	0.040	0.061	0.0093	-	-	> 45	-	> 45	-	-	-
Compound 11	0.013	0.018	0.012	-	-	9	-	> 45	-	-	-
Compound 12	0.0001	0.00047	0.000087	-	-	> 45	-	0.016	-	-	-
Compound 13	-	0.500	-	-	-	-	-	-	-	-	-

Structures are present in the tables corresponding to the molecules [9, 14, 19, 21–23, 42, 43, 45–50, 52, 56, 57, 60–68, 71, 73, 74, 78, 80–91]. *: Ki value reported in place of an IC_50_; NI: no inhibition observed; -: not applicable

## Abbreviations

FDA: Food and Drug Administration
FP: foot-pocket
HATs: histone acetyl transferases
HDACis: histone deacetylase inhibitors
HDACs: histone deacetylases
IC_50_: half maximal inhibitory concentration
Lys: lysine
PDB: Protein Data Bank
Phe: phenylalanine
PK: pharmacokinetic
TSG: tumor suppressor gene
ZBG: zinc-binding group
Zn: zinc

## Declarations

### Copyright

© The Author(s) 2023.
